# Biomarkers of Guillain-Barré Syndrome: Some Recent Progress, More Still to Be Explored

**DOI:** 10.1155/2015/564098

**Published:** 2015-09-16

**Authors:** Ying Wang, Shuang Sun, Jie Zhu, Li Cui, Hong-Liang Zhang

**Affiliations:** ^1^Neuroscience Center, Department of Neurology, The First Hospital of Jilin University, Changchun 130000, China; ^2^Department of Neurology, Heilongjiang Provincial Hospital, Harbin, China; ^3^Department of Neurobiology, Care Sciences and Society, Karolinska Institute, Stockholm, Sweden

## Abstract

Guillain-Barré syndrome (GBS), the axonal subtype of which is mainly triggered by *C. jejuni* with ganglioside-mimicking lipooligosaccharides (LOS), is an immune-mediated disorder in the peripheral nervous system (PNS) accompanied by the disruption of the blood-nerve barrier (BNB) and the blood-cerebrospinal fluid barrier (B-CSF-B). Biomarkers of GBS have been extensively explored and some of them are proved to assist in the clinical diagnosis and in monitoring disease progression as well as in assessing the efficacy of immunotherapy. Herein, we systemically review the literature on biomarkers of GBS, including infection-/immune-/BNB, B-CSF-B, and PNS damage-associated biomarkers, aiming at providing an overview of GBS biomarkers and guiding further investigations. Furthermore, we point out further directions for studies on GBS biomarkers.

## 1. Introduction

*Guillain-Barré Syndrome*. Guillain-Barré syndrome (GBS) is an immune-mediated disorder in peripheral nervous system (PNS) and experimental autoimmune neuritis (EAN) serves as a main animal model of GBS. GBS is typically triggered by antecedent infections and* C. jejuni* is blamed for at least one-third of these infections. Nevertheless, only one in 1,000–5,000 patients with* Campylobacter* enteritis will develop GBS [1, 2] and GBS patients with the same type of infection can have distinct clinical manifestations. Thus, both infection and host factors may influence the pathogenesis and the development of GBS.

The cardinal step in the development of GBS is exerted by the immune response. A subset of* C. jejuni* strains contains lipooligosaccharides (LOS), a kind of carbohydrate structure located on the outer membrane, which mimic the gangliosides in human. Autoantibodies that cross-react with gangliosides are provoked by antecedent infections and attack the PNS by activating complements [3]. Furthermore, the unbalance of Th1/Th2/Th17/Treg and M1/M2 is observed in both GBS and EAN [4]. Cytokines, chemokines, complements, and other immune- and inflammatory-associated factors are also proved to play an essential role in GBS and EAN [5]. Nerve biopsy studies demonstrate segmental demyelination and axonal degeneration as well as infiltration of macrophages, lymphocytes, and mast cells in the endoneurium of nerves in the PNS [6].

Damage to the PNS and the barriers, including the blood-nerve barrier (BNB) and the blood-cerebrospinal fluid barrier (B-CSF-B), is the pathological feature of GBS. BNB and B-CSF-B are barriers between blood and nerve/CSF that maintain a relatively stable environment to nerve/CSF. Distinct types of peripheral nerves damage address GBS as a highly diverse spectrum of clinical manifestations. A rapidly progressive, symmetrical weakness of the limbs in combination with hyporeflexia of areflexia is the clinical character of GBS [3]. Some of the GBS patients are also accompanied by cranial nerve involvement, sensory deficits and ataxia and may suffer from pain and autonomic dysfunction [3]. GBS is divided into two major subtypes: acute inflammatory demyelinating polyneuropathy (AIDP) and axonal subtypes including acute motor axonal degeneration neuropathy (AMAN) and acute motor and sensory axonal neuropathy (AMSAN). Nerve conduction studies (NCS) can help discriminate these subtypes of GBS in clinic.

*Overview of Biomarkers for GBS*. A biomarker is a characteristic that can be objectively measured and evaluated as an indicator of a physiological as well as a pathological process or pharmacological response to a therapeutic intervention. The diagnosis of the GBS is still challenging due to the lack of a single specific diagnostic test, which in some cases leads to a delay in the correct diagnosis and hence in the initiation of immunomodulatory treatment against GBS. The diagnosis of GBS is rather based on a combination of clinical features, NCS and analysis of the CSF at present [3]. However, the variety of clinical manifestations as mentioned above may mislead the diagnosis and NCS/CSF examination fails to show the abnormity at an early stage of the disease [3]. Lack of specific biomarkers that could eventually assist in the clinical diagnosis and in monitoring disease progression as well as the efficacy of immunotherapy has been a serious problem in GBS.

Serum, CSF, and peripheral nerve tissue are the main sources of biomarkers for GBS. It is noteworthy that the CSF is the most important source of biomarkers. Proximal nerve roots located in the subarachnoid region are floating freely in CSF and are in close contact with CSF. Therefore, the altered protein content of CSF could mirror the damage within the tissue of the nervous system [7]. Moreover, the dysfunction of B-CSF-B and BNB also permits CSF to serve as an essential source of biomarkers. B-CSF-B damage results in an alteration of CSF flow rate that modulates the protein content in CSF [8] and BNB lesion leads to the influx of serum proteins into the CSF [7]. Furthermore, the intrathecal synthesis of proteins also contributes to the changes of protein content in CSF.

At present, a growing number of studies focus on biomarkers in GBS. Although Johannes Brettschneider et al. first systemically reviewed the studies of GBS biomarkers published until October 2007, they only took CSF biomarkers into consideration. A panoramic review of biomarkers in GBS is still lacking. Herein, we summarize the studies of infection-, immune-, and BNB, B-CSF-B, and PNS damage-associated biomarkers in GBS to provide an outlook of GBS biomarkers.

## 2. Biomarkers of GBS (Table 1)

### 2.1. Infection-Associated Biomarkers

Only a subset of* C. jejuni* strains containing ganglioside-mimicking LOS could trigger GBS and the synthesis of LOS is controlled by a set of polymorphic genes and enzymes that vary greatly between different* C. jejuni* strains [3].

#### 2.1.1. LOS, Serotype, and Sequence Type of Campylobacter as Biomarkers

The gene contents of LOS loci are divided into eight classes (classes A to H). The expression of classes A, B, C, E, F, and H loci was found in GBS-associated* C. jejuni* [9, 10]. The Thr51 variant of* C. jejuni cst-II* gene that determined the structure of LOS was associated with the occurrence of GBS while the Asn51 variant was associated with MFS [11]. Moreover,* Campylobacter* strains with Penner heat-stable (HS) serotypes, including HS:1, HS:2, HS:4, HS:19, HS:23, and HS:41, were overrepresented among the strains isolated from GBS patients [2, 12, 13]. Furthermore, relatedness between sequence type 22 complex and GBS isolates was suggested [14].

#### 2.1.2. *C. jejuni* DNA-Binding Protein from Starved Cells (C-Dps)

A high level of C-Dps is produced to protect bacterial DNA from damage under the condition of oxidative or nutritional stress via specifically binding to the sulfatide that is important for the maintenance of the ion channels on myelinated axons and for paranodal junction formation. Recently, C-Dps was elucidated as a potential contributor to the peripheral nerve insult in GBS. After C-Dps was injected into the rat sciatic nerves, it densely binds to the myelin sheath and the nodes of Ranvier. And NCS disclosed a compound muscle action potential amplitude reduction [15]. Anti-C-Dps IgG was detected in* C. jejuni*-related GBS patients but not in healthy controls (HCs) or in patients with OIND. The frequency of production of anti-C-Dps IgG in* C. jejuni*-related GBS patients was significantly higher than that in* C. jejuni* enteritis patients without GBS (62.5% versus 9%). C-Dps was also found in serum of some* C. jejuni*-related GBS patients (14.8%) [16].

### 2.2. Immune-Associated Biomarkers

**Figure 1:**
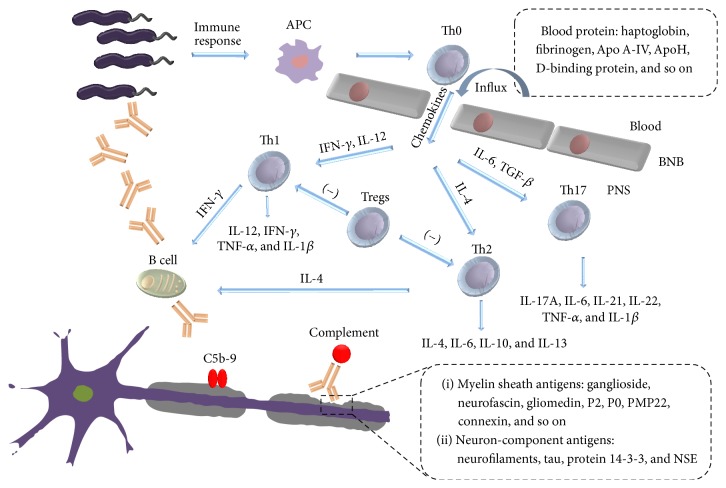
Axonal damage type of GBS is triggered by a subset of* C. jejuni* containing ganglioside-mimicking LOS on outer membrane. Immune response to* C. jejuni* induces the unbalance of Th1/Th2/Th17/Treg and cytokines that is crucial for the development of GBS. Chemokines are responsible for the infiltration of immune cells and complements activated by antibodies could mediate PNS lesion. Damage of barriers and PNS permits CSF to serve as an important source of biomarkers. Structural molecules of PNS, including myelin sheath molecules and neuron molecules, are released to CSF due to the damage of PNS and may provoke further immune response. Disturbance of BNB also results in an alteration of protein content in CSF due to blood protein influx.

LOS of* C. jejuni* activates the innate immune response via interacting with immunoglobulin-like receptor LMIR5, TLR 4, and sialic acid-specific receptors which are involved in the DC-mediated Th cell differentiation and B cell proliferation [17–19]. Furthermore, an intricate immune network has been addressed with a crucial role in the pathogenesis and the development of GBS [4] (Figure 1).

#### 2.2.1. Gene Polymorphisms

Fc*γ*R is a family of cell-surface molecules linking humoral and cell-mediated responses. It is expressed on almost all immune cells and plays a key role in defending against pathogens. It showed that the leukocyte degranulation and phagocytosis in GBS could be induced/blocked by anti-GM1 IgG/IVIg via Fc*γ*R that are localized on Schwann cells (SCs) and perineurial cells [20, 21]. Several studies have documented the relationship between Fc*γ*R/FcRL gene polymorphism and GBS. Among others, human leukocyte Fc*γ*R genes are divided into three classes: Fc*γ*RI, Fc*γ*RII, and Fc*γ*RIII. Fc*γ*RIIa, Fc*γ*RIIb, Fc*γ*RIIIa, and Fc*γ*RIIIb were found to be associated with the severity of GBS [22, 23]. The frequencies of expression of FcRL3-3-169C, FcRL3-6 intron 3A, and FcRL3-8 exon 15G alleles were significantly higher in GBS patients compared with HCs [24].

Studies on the relationship between the gene polymorphism of human leukocyte antigen (HLA) complex and GBS have elucidated that the frequencies of DQbeta 1^*∗*^060x, DRB^*∗*^03:01, DRB^*∗*^07:01, DRB^*∗*^01:01, DRB1^*∗*^14/DQB1^*∗*^05, and DRB1^*∗*^13/DQB1^*∗*^03 HLA genotypes were increased in GBS patients while the frequency of DR6 HLA genotype was elevated in control group [25–27]. Furthermore, HLA-DRB1^*∗*^01, HLA-DQA1^*∗*^0301, and HLA-DQA1^*∗*^0302/HLA-DQB1^*∗*^03 were found to be related to mechanical ventilation, anti-GM1 IgG levels, and recent* C. jejuni* infection, respectively [28–30]. DQBRLD^55–57^/ED^70-71^ epitopes, DR*β*E^9^V^11^H^13^ epitopes, and HLA-DRB1^*∗*^1301 allele were found to be associated with the development of AIDP while the DQ*β*RPD^55–57^ epitopes were found to be associated with the protection from AIDP [31, 32]. In AMAN patients, the frequencies of HLA-DRB1^*∗*^1301-03 and HLA-DRB1^*∗*^1312, taken collectively, were increased [32].

Gene polymorphisms of other important molecules in GBS were studied as well. It has been documented that CD1A^*∗*^01/02 and CD1E^*∗*^01/02 genotypes, A(-670)G single nucleotide polymorphism in the promoter region of CD95, TNF-*α*308 G/A and 857 C/T polymorphisms, mannose-binding lectin H allele/HY promoter haplotype/HYA haplotype, macrophage mediators SNPs, TCR V*β* and V*δ* genes, CD95 A(-670)G SNP, TLR 4 Asp299Gly polymorphism, killer-immunoglobulin-like receptor genotype, and glucocorticoid receptor genotype were related to GBS [28, 33–39].

#### 2.2.2. Cytokines

Cytokines are polypeptides that play a fundamental role in initiating, propagating, and regulating tissue-specific autoimmune injury (Figure 1). They act as signal molecules in an autocrine or paracrine fashion between cells of the immune system.

IFN-*γ* has a dual role in GBS. On the one hand, to date, most studies have addressed IFN-*γ* with a proinflammatory role in EAN and GBS. Clinical disability of GBS patients was positively correlated with the elevated serum level of IFN-*γ* and was improved after treatment with intravenous immunoglobulin (IVIg) [40]. On the other hand, recent evidence that IFN-*γ* could convert peripheral CD4(+)CD25(−) T cells to CD4(+)CD25(+) regulatory T cells in GBS [41] and IFN-*γ* deficiency exacerbated EAN via upregulating Th17 cells despite a mitigated systemic Th1 immune response [42] defined an anti-inflammatory role for IFN-*γ*.

It seems that TNF-*α* plays a dual role in GBS as well. The proinflammatory function of TNF-*α* was identified by the association between the increased serum TNF-*α* levels and the severity of GBS [40]. In addition, treatment with IVIg significantly reduced the levels of plasma TNF-*α* and TNFR1 while enhancing the levels of sTNFR1—an antagonist of TNF-*α* [40, 43]. However, TNFR2 might have a protective role in GBS as well. As demonstrated in another study, serum TNFR2 was increased after IVIg treatment in patients with AMAN [44]. It was demonstrated that TNF-*α* secretion was associated with the altered balance of different subtypes of macrophages in EAN [45].

IL-17 and IL-22 secreted by Th17 cells play a critical role in inflammatory disease and mucosal host defense. In GBS, plasma IL-17A and IL-22 levels were markedly elevated during the acute phase and the IL-17A concentration was reduced after IVIg therapy [46]. Both plasma and CSF IL-17A levels were positively correlated with the GBS disability scale scores [40]. In EAN, IL-17A expressed in the sciatic nerves was significantly downregulated by AUY954 treatment [47]. Administration of recombinant IL-17A provoked the infiltration of inflammatory cells into the sciatic nerves and induced more severe demyelination in EAN [48].

IL-18 was overexpressed in the nerve roots of EAN animals [49]. EAN mice exhibited attenuated clinical severity and impaired Th1 response in inflamed nerves when treated with anti-IL-18 monoclonal antibody [50]. However, more recent study elucidated that IL-18 deficiency in EAN inhibited the production of IFN-*γ*, TNF-*α*, and IL-10 but not the clinical severity. It indicated that IL-18 may act as a coinducer of Th1 and Th2 cytokines in EAN [51]. Preliminary data support elevation of IL-18 levels in serum of GBS patients [49].

For other cytokines, IL-1*β* was immunolocalized on the membranes of SCs in sural nerves [52] and IL-1*β* was detected in the CSF of GBS patients. For anti-inflammatory cytokine IL-4, its upregulation in the recovery phase defined it with a role in terminating EAN and GBS [53, 54]. Similarly, IL-10 also helped terminate GBS/EAN; however, it might worsen the disease by promoting the generation of anti-ganglioside antibodies [55–57]. IL-6 was found to be upregulated in serum and CSF of GBS patients [58, 59]. Intraneural injection of recombinant rat IL-6 induced high inflammation and severe demyelination in EAN [60]. IL-12 was reported to have a major role in the initiation, enhancement, and perpetuation of pathogenic events in both EAN and GBS by promoting Th1 cell-mediated immune response while suppressing the Th2 response [44, 61]. IL-16 was suggested to be a pathological contributor to EAN due to a strong correlation of IL-16+ cell accumulation with local demyelination in perivascular areas of sciatic nerves [62]. IL-23 might play a cardinal role during the early and acute phase of EAN. IL-23 was detectable in CSF samples of GBS patients [5]. The plasma and CSF levels of IL-37 in GBS patients at the acute phase were significantly higher than HCs, and treatment with IVIg significantly reduced the serum levels of IL-37 [40]. Interestingly, TGF-*β*1 levels in plasma were decreased at the onset of GBS [63] while they were increased during the recovery phase [64]. However, the number of TGF-*β* secreting cells was elevated in all phases of GBS [64].

Additionally, some cytokines such as IL-21, IL-27, and IL-35 that play an important role in other autoimmune diseases may be the biomarkers for GBS. Further studies are needed to explore their possible roles in GBS.

#### 2.2.3. Complements

Complements are another group of candidates for GBS biomarkers (Figure 1). In the presence of complements, serum from GBS patients exhibited demyelinating activity both in vitro and in vivo [65, 66]. A growing body of evidence pointed out that complements activated by the anti-ganglioside autoantibodies disrupted sodium channel clusters, paranodal axoglial junctions, the nodal cytoskeleton, and microvilli of SCs in GBS [67]. Furthermore, the blockade of complements activation by IVIg treatment or anti-complements antibodies prevented the formation of membrane attack complex (C5b-9) and the emergence of clinical signs [68, 69].

The presence of C3 in PNS was observed in both GBS and EAN. A proteomic study addressed an enhanced C3 level in CSF [70]. Complement activation marker C3d was localized on the outer surface of the SCs in GBS patients and C3d-positive fibers were found with vesicular changes on the outermost myelin lamellae [71]. C3d binds to the nodal axolemma of motor fibers in AMAN and to the myelinated internodes in more severe cases [72]. Similar results appeared in animal studies [73]. C5b-9 activated by anti-ganglioside antibodies mediated a direct injury to peripheral nerves. SC5b-9, an inactive isoform of C5b-9, was detected in both serum and CSF of GBS patients [74, 75]. Deposits of C5b-9 on SCs, myelin sheaths, macrophages, and endothelial cells were shown in GBS and EAN as well [76]. Notably, deleterious effects of complements could be prevented by eculizumab which blocked the formation of human C5a and C5b-9 [68].

#### 2.2.4. Chemokines

Chemokines are low-molecular-weight (8–14 kDa) cytokines that are involved in the directed migrations (chemotaxis) across concentration gradients and the activation of immune cells. Chemokines are classified into 4 subfamilies based on the organization of two conserved cysteine residues: CC, CXC, CX3C, and C subfamilies. Multiple lines of evidence point out that the chemokines are involved in the immune response of GBS patients.

CCL2 was expressed on SCs, infiltrating cells and blood vessels with its receptor CCR2 expressed on macrophages and lymphocytes. CCL2, the secretion of which was stimulated by TNF-*α*, was postulated to facilitate the trafficking of autoreactive leucocytes across the BNB in GBS [77]. High expression of CCL2 and CCR2 was observed in the sciatic nerves of severe EAN [78]. In GBS, the circulating CCL2 levels were elevated at the acute phase and peaked at the time of plateau but normalized at the recovery stage [79].

The peak number of CCL3 positive cells was seen in the sciatic nerves of EAN 14 days after onset and was correlated with the severe clinical presentations. Anti-CCL3 antibody was demonstrated to attenuate the severity of EAN and inhibit the inflammation and demyelination in sciatic nerves. CCR1 and CCR5 expressed by endoneurial macrophages with CCL5 colocalizing to axons were increased on sciatic nerves of EAN and GBS [78, 80]. The levels of CX3CL1/CX3CR1 were higher in dorsal horns of EAN and CX3CL1 CSF/serum ratios were observed to be elevated in GBS [81, 82]. The number of CXCL2 positive cells reached a maximum of 21 days after immunization; however, anti-CXCL2 antibody failed to diminish the clinical severity of EAN [83]. CXCL10 was localized on the endoneurial endothelial cells and within the endoneurial interstitium, with its receptor CXCR3 on lymphocytes [78]. CXCL10/CXCR3 levels were significantly increased in the sciatic nerves of EAN [78] and the enhanced CSF levels of CXCL10 were measured in GBS patients [84]. The median CSF concentrations of CCL7, CCL27, CXCL9, and CXCL12 were also higher in GBS [84].

Additionally, other immune-related biomarkers are listed in Supplementary Table 1 (in Supplementary Material available online at http://dx.doi.org/10.1155/2015/564098). 

### 2.3. BNB, B-CSF-B, and PNS Damage-Associated Biomarkers

#### 2.3.1. BNB and B-CSF-B Damage-Associated Biomarkers

The protein content in CSF is altered in GBS patients due to B-CSF-B/BNB disturbance and intrathecal synthesis of proteins. 80% of the proteins in CSF are blood derived with the other 20% being brain derived. There are mainly three types of brain-derived proteins in CSF with different sources, including protein originating from neurons and glial cells, proteins released from leptomeninges, and proteins with a nonnegligible blood-derived fraction in CSF [8]. A reduced CSF flow rate caused by B-CSF-B dysfunction influences the molecular flux in CSF [8]. BNB insults permit circulation proteins influx into CSF. Intrathecal synthesis of proteins obviously contributes to the protein content alteration in CSF and it could be measured with CSF index of protein x. CSF index of protein x is calculated as (CSF level of protein x/plasma level of protein x)/(CSF level of albumin/plasma level of albumin). Although the alterations of many biomarkers mentioned above and PNS damage-associated biomarkers discussed in the next subtitle are also due to the damage of barrier, we will review some other barriers damage-associated biomarkers in this section.

*(1) Brain-Derived Proteins*. A combination of elevated total protein (TP) level and normal cell counts in the CSF may be regarded as the first CSF biomarker for GBS. The elevation was directly associated with the time point of performing lumbar puncture [85].

Prealbumin is synthesized predominantly by parenchymal cells of the liver and then secreted into the plasma. However, the prealbumin in CSF originates mainly (90%) from the choroid plexus in the ventricles. The levels of the prealbumin in both plasma and CSF were elevated in patients with GBS; nevertheless, the CSF index of prealbumin was decreased [86]. In contrast, another study elucidated that CSF prealbumin levels were reduced at admission and were associated with greater clinical severity [87]. Transthyretin, the former prealbumin, in CSF originates predominantly from the choroid plexus; however, a small blood-derived fraction of about 10% can be calculated to contribute to the concentration in CSF. The study of transthyretin in CSF showed controversial results. Proteome study demonstrated downregulated levels of transthyretin [70] while ELISA analysis reported upregulated levels [88].

S100B is a calcium-binding protein originating from glial cells. It was reported to be upregulated in the CSF of GBS patients and was correlated with the GBS disability scale scores in AIDP as well as with months to recovery [89, 90]. Cystatin C is produced by all nucleated cells and is primarily secreted from choroid plexus into CSF. Cystatin C in CSF can be regarded as brain derived with no more than 1% from serum. It was proved by both ELISA and proteome study that cystatin C levels were decreased in CSF of GBS patients [91, 92]. Prostaglandin D(2) synthase is an abundant brain protein in CSF and it is tied closely with inflammatory processes. The concentration of prostaglandin D(2) synthase was significantly increased in the CSF, whereas the intrathecal synthesis was significantly decreased in AIDP patients [93]. Hypocretin-1 is a hypothalamic originated neuropeptide. The levels of hypocretin-1 were moderately downregulated in early stage of GBS and were associated with central nervous system abnormalities [94].

*(2) Blood-Derived Proteins*. Haptoglobin is a plasma protein with haemoglobin-binding capacity and is a positive acute-phase protein that functions as an inhibitor of prostaglandin synthesis and angiogenesis. Proteomic study revealed that the expression of haptoglobin was enhanced in CSF of GBS patients [91]. This result was consistent with the result of another study using ELISA [86]. Fibrinogen is a plasma glycoprotein involved primarily in the blood clotting cascade. Preliminary data of fibrinogen concentration in CSF was contradictory. One study demonstrated decreased concentrations of fibrinogen [95], whereas the other reported elevated levels with decreased CSF index of fibrinogen [86]. Additionally, proteomic studies also demonstrated enhanced Apo A-IV, ApoH, vitamin D-binding protein, and *α*-1-antitrypsin levels in CSF of GBS patients [70, 91, 95].

#### 2.3.2. PNS Damage-Associated Biomarkers (Figure 1)

*(1) Myelin Sheath-Associated Markers.* Gangliosides are a group of glycosphingolipids characterized by the presence of one or more sialic acid residues in the oligosaccharide chain. Anti-ganglioside antibodies are often closely associated with clinical phenotypes and specific clinical signs of GBS. This association is likely to depend upon the diverse distribution of ganglioside antigens in the PNS. The antigens targeted are located at or near the nodes of Ranvier in AMAN and on myelin sheath in AIDP. The antibodies can activate complements that provoke the formation of C5b-9. Interestingly, excepting antibodies against single gangliosides, patients can also have antibodies against the combination of epitopes from ganglioside complexes (GSCs). Such complexes are located in specialized microdomains or “lipid rafts” in the cell membranes [3]. In total, IgG and IgM were the most frequent types of antibodies to ganglioside [109]. Seropositive patients were more frequently involved with preceding diarrhea and pure motor neuropathy [109, 110]. IgG1 was related to diarrhea and poor outcomes while both IgG1 and IgG3 were related to upper respiratory tract infections and better outcomes [110]. Associations between antecedent infections, subtypes of GBS, clinical manifestations, and the types of antibodies to ganglioside are listed in Table 2.

Neurofascin and gliomedin are neuronal cell adhesion molecules that play a central role in the formation of nodes of Ranvier and are considered as novel target antigens in GBS. Investigations reported the detectable autoantibodies to neurofascin and gliomedin in both GBS and EAN [111, 112]. In EAN, it is pointed out that the immunity to neurofascin and gliomedin was prior to the demyelination. They also induced progressive neuropathy characterized by the deposition of autoimmune antibodies and the defects of conduction [112, 113].

P2, P0, PMP22, and connexin 32 are peripheral myelin proteins, and both P2 and P0 are used to induce EAN. Antibodies to P0, PMP22, P2_14–25_, and connexin 32 were detected in GBS patients [114–116]. *α*6*β*4 is a laminin receptor that mediates the recognition and attachment to extracellular matrix proteins in SCs and myelin. *α*6*β*4 immunoreactivity was detected in 66% of GBS patients [117]. Anti-phospholipid antibodies were detected in GBS patients and were downregulated by IVIg [118]. Patients with Gal-C-GBS were more frequently involved with sensory deficits, autonomic dysfunctions, and antecedent* Mycoplasma pneumonia* infections [119].

*(2) Neuron-Component-Associated Biomarkers*. Neurofilaments are cytoskeletal proteins that are particularly abundant in large myelinated axons. Their release into the CSF addressed them as a promising biomarker for neurodegeneration. The CSF neurofilaments levels were increased in GBS patients, positively correlated with the GBS disability scale scores in AMAN, and predicted the clinical/electrophysiological outcomes of GBS [89, 120]. Moreover, enhanced neurofilament levels were seen in the corresponding serum samples as well [121].

Tau proteins modulate assembly and stability in axonal damage marker. Tau concentrations in CSF from GBS patients were enhanced. The elevated tau levels were correlated with the GBS disability scale scores in AMAN and predicted poor clinical outcomes [89]. 14-3-3 proteins are highly conserved acidic polypeptides that are particularly abundant in the nervous system. 14-3-3 protein assay showed that 14-3-3 expressed by mononuclear inflammatory infiltrates and SCs was detected as early as 12 to 48 hours after disease onset [108]. Neuron-specific enolase is a glycolytic enzyme predominantly presenting in neurons and neuroendocrine cells. Neuron-specific enolase was significantly elevated in CSF of GBS patients and was correlated with months to recovery [90].

Additionally, other biomarkers that have not been included above but are related to GBS are presented in Supplementary Table 2.

## 3. Concluding Remarks

Specific biomarkers are still lacking to help make exact diagnosis and predict the outcomes of GBS. A growing number of studies focus on the biomarkers of GBS and address infection-, immune-, and BNB, B-CSF-B. and PNS damage-associated molecules as potential biomarkers for GBS. Serum, CSF, and peripheral nerves are the main sources of biomarkers. Many of these biomarkers are proved to be associated with the pathogenesis, development, and recovery of GBS. IVIg treatment, inhibiting the Fc-mediated activation of immune cells as well as the binding of autoimmune antibodies to their targets, is one of the first-line immunotherapies of GBS. IVIg downregulated Th17, Th22, IL-17, and IL-22 in GBS patients and mediated expansion of regulatory T cells [46, 122]. Clinical improvement with prominent peripheral mobilization of HLA-DR^high^CD138^low^CXCR4^low^ immature plasma cells was also observed in GBS patients. Further studies are warranted to explore more therapeutic biomarkers in GBS [123]. However, the related studies and the using of biomarkers have limitations. Firstly, several biomarkers, described as “predisposition” or “susceptibility indicators,” such as gene polymorphisms, may not be used in clinic as a basis for predictive testing, because they are found too frequently in healthy populations and may not develop the suspected diseases, although they may have a higher statistical probability that a disease may occur. Secondly, some biomarkers are discovered by flawed methods and their clinical value is negligible. A standard protocol to measure the biomarkers has not been established yet and the studies using distinct methods casually acquire conflicting results. The disease-related proteins in CSF may be produced intrathecally; regretfully, most of the studies fail to use CSF index to evaluate the intrathecal synthesis. Furthermore, the annual incidence of GBS is as low as 1-2/100,000 and most of the investigations have a relative small sample size. Numbers of GBS patients included are generally too low to permit a final verdict. Last but not the least, the clinical applications of many biomarkers may be withdrawn by expensive methods, invasive examinations, low sensitivity/specificity, and so forth. Further investigations are needed to continue searching for new biomarkers and studying existing biomarkers for GBS.

## Supplementary Material

Supplementary Table 1: An intricate network of immune system plays an important role in the pathogenesis and the development of GBS. Except the classic immune factors including cytokines, complements and chemokines, many other molecules are found involving in the pathogenesis of GBS due to their immune effect, including erythropoietin, heat shock protein, apolipoprotein E, C-reactive protein, neopterin, matrix metalloproteinases, reactive oxygen species, cell adhesion molecules, microRNA-155 and osteopontin. They are described in detail in the table.
Supplementary Table 2: Some of the biomarkers are beyond the classification set in the review. Additional information of them are provided in the table.

## Figures and Tables

**Table 1:** Biomarkers in GBS.

Classification of biomarkers	Biomarkers
Infection-associated biomarkers	LOS, serotype and sequence type of *Campylobacter jejuni* DNA-binding protein
Immune-associated biomarkers	
Gene polymorphisms	Fc*γ*R, HLA, CD1, CD95, TNF-*α*, mannose-binding lectin, macrophage mediators, TCR, TLR4, killer-immunoglobulin-like receptor, glucocorticoid receptor
Cytokines	IFN-*γ*, TNF-*α*, IL-17, IL-22, IL-18, IL-1*β*, IL-10, IL-6, IL-12, IL-16, IL-23, IL-37, TGF-*β*1
Complements	C3, C5b-9, C5a
Chemokines	CCL2, CCL3, CX3CL1, CXCL2, CXCL10, CCL7, CCL27, CXCL9, CXCL12
Others	Erythropoietin, heat shock protein, apolipoprotein E, C-reactive protein, neopterin, matrix metalloproteinases, reactive oxygen species, cell adhesion molecules, microRNA-155, osteopontin
BNB/B-CSF-B damage-associated biomarkers	
Brain-derived proteins	Total protein, prealbumin, transthyretin, S100B, cystatin C, prostaglandin D(2) synthase, hypocretin-1
Blood-derived proteins	Haptoglobin, fibrinogen, Apo A-IV, ApoH, vitamin D-binding protein, *α*-1-antitrypsin
PNS damage-associated biomarkers	
Myelin sheath-associated biomarkers	Autoantibodies to ganglioside, neurofascin, gliomedin, P0, PMP22, P2_14–25_, connexin 32, *α*6*β*4, phospholipid
Neuron-component-associated biomarkers	Neurofilaments, tau proteins, 14-3-3 proteins, neuron-specific enolase
Other biomarkers for GBS	Creatine kinase heparin sulfate glycosaminoglycans, glial fibrillary acid protein, triglyceride and hyponatremia

**Table 2:** Autoantibodies to gangliosides and their association with GBS.

Antigen	Infection	Subtype	Association with GBS	Reference
GA1		AMAN		[96]

GD1a			Younger, predominantly male, facial nerve involvement	[97]

GD1b			Reversible conduction failure, ataxia	[96, 96]

GalNAc-GD1a	G^a^	Axonal dysfunction	Distal weakness, low amplitudes for the compound muscle action potentials, facial palsy	[98]

9-O-Acetyl GD1b			Potential target	[99]

GD3			Ophthalmoparesis	[100]

GM1	G	AMAN	Reversible conduction failure, rapidly progressive stage, distal distribution of weakness, not sensitive to plasma change treatment	[96, 97]

GT1a			Ophthalmoplegia	[97]

GT1b			Ataxia	[101]

GT3			Ophthalmoparesis	[100]

*O*-Acetyl GT3			Ophthalmoparesis	[100]

GQ1b			Ataxia, ophthalmoparesis	[101, 102]

LM-1			Potential target	[103]

GD1a/GD1b			Severe disability, mechanical ventilation	[104, 105]

GD1b/GT1b			Severe disability, mechanical ventilation	[104, 105]

GM1/GalNac-GD1a	R^b^		Pure motor GBS, conduction blocks at intermediate nerve	[106]

GM1/PA			Potential target	[107, 108]

GM1/GD1a			Potential target	[107]

GM1/GT1b			Potential target	[105]

LM1/GA1		AMAN	Reversible conduction failure	[96]

^a^Gastrointestinal infection.

^b^Respiratory infection.
