# Supplementary material for: Biomarkers of Guillain-Barré Syndrome: Some Recent Progress, More Still to Be Explored
Source: Mediators Inflamm. 2015 Sep 16;2015:564098. doi: 10.1155/2015/564098 (PMC4588351; doi:10.1155/2015/564098)
Supplement: Supplementary file 1 — Supplementary Table 1: An intricate network of immune system plays an important role in the pathogenesis and the development of GBS. Except the classic immune factors including cytokines, complements and chemokines, many other molecules are found involving in the pathogenesis of GBS due to their immune effect, including erythropoietin, heat shock protein, apolipoprotein E, C-reactive protein, neopterin, matrix metalloproteinases, reactive oxygen species, cell adhesion molecules, microRNA-155 and osteopontin. They are described in detail in the table. Supplementary Table 2: Some of the biomarkers are beyond the classification set in the review. Additional information of them are provided in the table. [file 564098.f1.pdf]

Supplementary Table 1. Other immune-related biomarkers

Biomarkers	Description of immune function	Alteration or function in GBS
erythropoietin	a pleiotropic cytokine	Erythropoietin was up-regulated in EAN lesions and played a neuroprotective role in both GBS and EAN (85-87).
heat shock protein	antigen presentation, cross-presentation, inducing production of pro-inflammatory cytokines and promoting dendritic cell maturation	High IgG titers to heat shock protein27, 60, 70 and 90 were observed in CSF from GBS patients (88). Up-regulation of Hsp70 that correlated with disease severity was observed in sciatic nerves of EAN. In CSF of GBS patients, high IgG levels to Hsp 70 and low Hsp70 levels were reported (88-91). αB-crystallin, a small heat shock protein was overexpressed in dorsal ganglia/spinal roots and IgG/IgM against αBC were found in CSF of GBS patients (92, 93).
apolipoprotein E	suppression of T cell proliferation, regulation of macrophage function, facilitation of antigen presentation and modulation of inflammation/oxidation.	Decreased ApoE levels were found in CSF of GBS patients and were proved to exaggerate GBS (94, 95). Further study revealed the isoform-specific effects of apoE in GBS (96, 97).
C-reactive protein	biomarker for infectious and inflammatory conditions	High basal levels of C-reactive protein were detected in GBS patients (98).
neopterin	a product of macrophages in response to lymphocytic activation	Circulation neoptrien was increased and related to the severity of GBS (99).
matrix metalloproteinases	disintegrate basement membrane and promote the transmigration of inflammatory cells	Expression of matrix metalloproteinases-2 was related to the degree of myelination in vitro and was detectible in the CSF of GBS patients (100). Circulation matrix metalloproteinases-9 was higher/lower in the acute phase/ recovery phase and correlated with disease severity, electrophysiologic abnormalities, lymphocyte penetrating capacities and TNF-α/IL-1β levels (35, 100-102).

reactive oxygen species	form a pivotal part of the innate immune defence against microorganisms	An imbalance among the levels of antioxidant activity and malondialdehyde activity could be followed in GBS (103).
cell adhesion molecules	cell-cell and cell-matrix interactions, adhesion and migration of immune cells	Serum endothelial-leukocyte adhesion molecule-1 levels were higher in acute phase and temporally correlated to disease severity (104, 105). Intercellular adhesion molecule-I and vascular cell adhesion molecule-1 levels were above normal values at progression and plateau, but tended to be normalized at recovery stage (84, 102, 106).
microRNA-155	central regulator of immune response	The expression of microRNA-155 was decreased in peripheral blood monocytes from GBS patients and the knockdown of microRNA-155 promoted the production of Th1-type cytokines in vitro (107).
osteopontin	'early T cell-activation gene 1'	Osteopontin levels were elevated in the CSF of GBS patients and were correlated with GBS disability scale scores (108).

Supplementary Table 2. Other biomarkers for GBS

Biomarkers	Alteration or function in GBS
creatine kinase	A case report described a GBS patient with cramping pain and elevated serum creatine kinase level (145).
heparin sulfate glycosaminoglycans	Serum IgM or IgG antibodies to heparin sulfate GAGs were observed in 34% of GBS patients while rare (1%) in controsl(146).
glial fibrillary acid protein	Serum GFAP levels were increased in GBS patients and were correlated with the GBS disability scale scores 6 months after neuropathy onset (147).
triglyceride	Decreased TG concentration was found in GBS patients (148).
hyponatremia	Facial weakness and mechanical ventilation in GBS patients were closely associated with hyponatremia. Hyponatremia was a predictor of poor outcomes as well (149).
